# Pediatric posttraumatic macular rupture

**DOI:** 10.22336/rjo.2025.21

**Published:** 2025

**Authors:** Hasimbegovic Selma, Stojanovic Andjela, Peric Miroslav, Tomic Zoran, Pidro Miokovic Ajla, Pidro Gadzo Aida

**Affiliations:** 1 Ophthalmology Department, General Hospital “Prim. Dr. Abdulah Nakas”, Sarajevo, Bosnia and Herzegovina; 2 Ophthalmology Department, Public Health Institution “Serbia”, East Sarajevo, Bosnia and Herzegovina; 3 Eye Hospital “Milos Clinic”, Belgrade, Serbia; 4 Ophthalmology Policlinic Vukas, Zagreb, Croatia

**Keywords:** traumatic macular rupture, pediatric ophthalmology, vitrectomy, TMR = traumatic macular rupture, BCVA = best corrected visual acuity, OCT = optical coherence tomography, PPV = pars plana vitrectomy, ILM = internal limiting membrane

## Abstract

**Objective:**

Macular rupture following ocular trauma is an uncommon but significant complication that can lead to vision loss. Due to the scarcity of literature on treatment options for traumatic macular ruptures (TMR) in pediatric patients, this case report aims to provide scientific insight and share our positive experience in treating a pediatric TMR.

**Materials and methods:**

A 6-year-old patient presented with reduced vision in the right eye following blunt trauma. Initial examination revealed hyphema, corneal edema, post-traumatic uveitis, and diminished visual acuity. Despite improvement in anterior segment findings, OCT confirmed a persistent full-thickness macular rupture three months post-injury, prompting surgical intervention.

**Results:**

After three months of observation to allow spontaneous closure, the patient underwent pars plana vitrectomy (PPV) with internal limiting membrane (ILM) peeling using the inverted flap technique and SF6 gas tamponade. OCT showed a closed macular rupture three months after surgery with residual tissue reorganization, and visual acuity improved from 0.02 to 0.3. The surgical approach resulted in successful anatomical closure and moderate functional improvement.

**Discussion:**

This case report highlights the successful management of a pediatric traumatic macular rupture (TMR) using pars plana vitrectomy (PPV) with internal limiting membrane (ILM) peeling. Despite the limited literature on the optimal treatment for TMR in children, the surgical approach described resulted in anatomic closure and functional improvement in the patient. The report emphasizes the importance of individualized treatment, considering both conservative and surgical options, particularly in cases where spontaneous closure is unlikely or delayed.

**Conclusion:**

TMH management in pediatric patients poses unique challenges due to a lack of standardized treatment protocols. While conservative observation is acceptable in cases with a higher chance of spontaneous closure, surgical intervention should be considered for more significant or persistent ruptures.

## Introduction

A macular rupture following ocular trauma (TMR) is a rare but significant complication that can lead to considerable vision loss [1]. While macular ruptures are most commonly degenerative, post-traumatic macular ruptures are relatively uncommon [1]. TMR is more common in young males and is usually caused by blunt trauma [2]. Unlike degenerative ruptures, which develop gradually over time, traumatic macular ruptures tend to occur suddenly, often associated with a rapid decline in visual acuity. The exact mechanism behind the formation of these ruptures is not fully understood; however, several theories exist regarding their pathogenesis. One theory suggests that the force transmitted during a blunt trauma leads to the expansion of the equator and the flattening of the posterior pole, which may result in rupture [3]. Another theory involves the sudden separation of the vitreous from the retina, leading to the rupture of the macula [4]. They are most commonly accompanied by other ocular pathologies, such as anterior or posterior hemorrhages, choroidal rupture, and others [2].

The first TMH was reported in 1869 by Donald Gass, who developed a staging system for grading idiopathic macular holes. However, this system cannot be applied to TMH, which are less frequent, with an incidence of 0.15-1.4% in open and closed globe injuries, respectively [1].

Once a post-traumatic macular rupture occurs, the next question is how to manage it. It is known that unlike idiopathic macular holes, TMH can often close spontaneously. Many vitreoretinal surgeons opt for a conservative approach, waiting for the rupture to close spontaneously. Some studies have reported positive outcomes with this method. In contrast, others suggest that surgical intervention may be necessary, with the timing of surgery varying depending on the surgeon’s experience and the specific case. Literature has documented both approaches, and the decision is often made on a case-by-case basis [1]. Since pediatric TMH is rarely reported, limited information is available about its treatment. Thus, we report a case of pediatric TMH and its treatment to provide our experience in this field of ophthalmology.

## Materials and methods

**Fig. 1:**
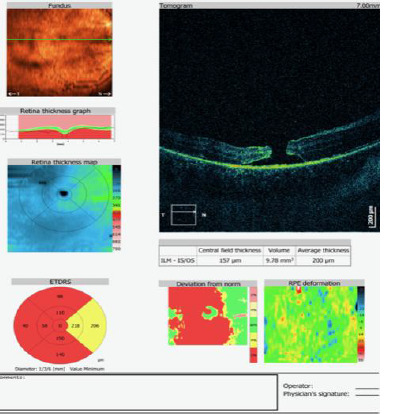
OCT examination after the injury

**Fig. 2:**
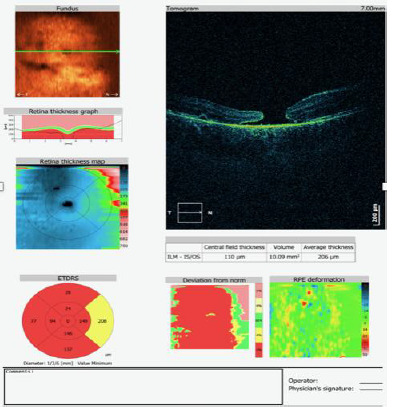
OCT examination 2 months after the injury

A 6-year-old patient presented with a sudden decrease in vision in the right eye following blunt trauma to the globe. The best-corrected visual acuity (BCVA) in the right eye was 0.02, and 1.0 in the left eye. Examination of the anterior segment revealed hyphema, corneal edema, signs of post-traumatic uveitis, and a diminished red reflex. On the sixth day of hospitalization, despite the regression of the anterior segment findings, BCVA in the right eye remained at 0.02. Indirect fundoscopy revealed a full-thickness macular rupture, which was confirmed by optical coherence tomography (OCT) (**Fig. 1**). A consultative evaluation by a vitreoretinal surgeon was conducted. Regular monitoring of the macular rupture was recommended three months after the injury. If the rupture persisted, vitrectomy with gas tamponade was considered. Two months after the injury, the patient’s BCVA in the right eye remained unchanged, and a follow-up OCT also showed a stable full-thickness macular rupture (**Fig. 2**).

## Results

Three months after the trauma, the macular condition remained unchanged. Subsequently, the patient was referred to the vitreoretinal surgeon, who performed pars plana vitrectomy (PPV) with internal limiting membrane (ILM) peeling using the inverted flap technique and 20% SF6 gas tamponade.

For pediatric patients undergoing vitrectomy, preoperative preparation generally includes thorough anesthesia evaluation and meticulous planning, as children may have higher risks for anesthesia-related complications. After the patient underwent an anesthesiology examination, the procedure was explained in detail to the parents, and informed consent for the surgery was obtained.

After sterilizing the surgical field and placing a sterile drape, a speculum was used to keep the eye open. Three small incisions were created in the pars plana, approximately 4 mm behind the limbus. These incisions are made to introduce instruments: a light source, an infusion line, and a vitrectomy probe. A vitrectomy probe was used to remove the vitreous body, which provided better access to the macula. Removing the vitreous is essential to relieve any traction on the macular hole that could interfere with healing. After a vitrectomy, a dye is applied to stain the internal limiting membrane (ILM), facilitating better identification of the ILM. The ILM was carefully lifted and removed around the macular hole using microforceps. The goal of ILM removal is to reduce traction in the macular area, which can aid in the healing of the hole. With the inverted flap technique, part of the ILM was not entirely removed. Instead, the ILM was dissected but left attached to the edges of the macular hole. The portion of the ILM still attached at the edge of the macular hole was carefully inverted and placed over the hole itself, creating a “flap”. This flap acts as a “scaffold” covering the hole, facilitating tissue regeneration. The inverted flap technique helps close the hole by allowing cell migration and the formation of retinal tissue over the ruptured area. At the end of the surgery, SF6 was injected into the vitreous cavity, creating pressure on the macula and holding the flap in place, which helps seal the hole. The gas was gradually reabsorbed over the following weeks. After surgery, the patient was advised to maintain a specific head position to ensure optimal gas pressure on the macula and to stabilize the flap during the healing process.

The patient was monitored postoperatively to assess the success of the surgery and the healing of the macula. Topical antibiotic and steroid eye drops were prescribed to reduce inflammation and the risk of infection. Regular follow-ups, including optical coherence tomography (OCT), were recommended to evaluate the closure of the hole and tissue organization. It was explained that postoperative head positioning and activity restrictions were crucial for successful healing.

**Fig. 3:**
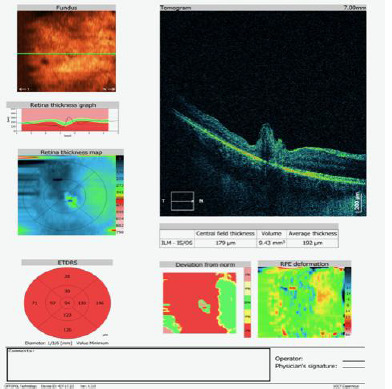
OCT examination 7 days postoperatively

**Fig. 4:**
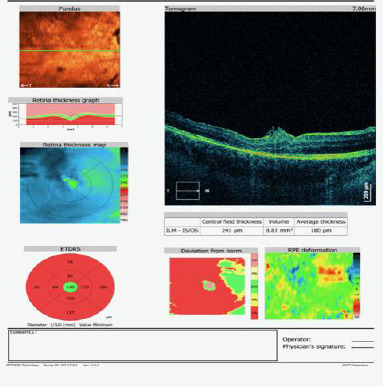
OCT examination 3 months postoperatively

The patient was followed up for three months postoperatively, and OCT imaging (**Fig. 3, 4**) showed a closed macular defect and residual postoperative tissue reorganization. The best-corrected visual acuity improved to 0.3 three months postoperatively.

## Discussion

Pediatric TMH is a rare clinical entity that presents a significant challenge for every ophthalmologist, particularly in its management, due to the lack of a standardized treatment protocol. TMH most commonly results from a blunt trauma, as was in our case report.

The mechanism of TMH involves the stretching of retinal tissue after a blunt trauma. Most researchers recommend a conservative treatment approach during the acute phase, as some TMHs have been shown to undergo spontaneous closure. However, in cases of more significant or persistent ruptures, the likelihood of spontaneous closure decreases, necessitating surgical intervention. In our case, there was no improvement three months after the injury, so a surgical approach was the only option to preserve vision. Often, after the surgery, only anatomic closure is achieved with no or minimum functional recovery due to underlying macular damage induced by trauma. Our case aligns with findings from similar studies, which report a small but significant improvement in visual acuity post-surgery [5].

Wendel et al. were the first to describe a surgical protocol for TMH closure, which consisted of five steps: PPV, induction of posterior vitreous detachment, ILM peeling with or without the inverted flap technique, fluid-gas exchange, and head-up positioning 7 days after surgery [6]. They have demonstrated that a higher success rate was achieved with surgical intervention performed within 6 months post-injury [6]. Following their report, other researchers have attempted to improve these results by adding surgical adjuvants, such as TGF-beta 2, serum, and platelet concentrate, into the hole after air exchange. A tamponade agent was used to finish the surgery. The goal of these agents was to assist in chorioretinal adhesion formation, thereby preventing further fluid from entering the subretinal space and, over time, facilitating TMH closure [7].

Both Wu et al. and Wachtlin et al. have reported successful surgical outcomes after PPV with internal limiting membrane peeling and gas tamponade, resulting in an improvement in visual acuity by two or more lines [5,8]. Kuhn et al. were among the first to describe PPV with ILM peeling in the treatment of TMH, achieving a 100% success rate with a visual acuity improvement of at least two lines in 94% of patients [9]. PPV with ILM flap was first proposed by Michalewska et al., achieving a 100% anatomical success rate and a significant visual improvement of 2 lines [10]. Similar improvement was found in studies by Rubin et al., and Garcia-Arumi et al., who reported 92% and 93% success rate with pars plana vitrectomy, ILM peeling, gas exchange and instillation of recombinant transforming growth factor beta-2 or instillation of 0.1 ml if platelet concentrate over the macular hole, respectively [11,12]. These similar results showed that there is no need for adjunctive therapy after PPV and ILM peeling with or without an inverted flap. Most of these studies showed improved visual acuity by at least two Snellen lines, aligning with our research.

There are some cases of spontaneous closure of TMH, where all of them were closed within one year of trauma and 55% within three months of the trauma [13,14]. Most were smaller, and most were without PVD [15,16]. A suggested mechanism for spontaneous closure involves the proliferation of glial cells, which promotes closure. Also, with the resolution of Berlin’s edema, the edges of the hole can return closer to each other, resolving a defect [17]. This is why some authors decide to wait at least three months for a spontaneous closure, as was the case in our report. Most of these holes were small, and it is believed that large holes result from higher blunt trauma and higher energy, which lead to more severe damage to the retina, RPE, and subretinal bleeding, retinal necrosis, and tissue loss, which prevents the macula from undergoing a complete reparative process [1].

There are two treatment options: observation and a surgical approach. Analyzing other studies, it can be concluded that waiting for the acute phase to pass is acceptable to allow for spontaneous closure of the rupture. However, this period should not be too prolonged, as earlier surgical intervention leads to better anatomical outcomes and visual acuity. The choice of surgical technique should be the one that is most suitable for the surgeon and with which they feel the most confident, as studies show that success rates and visual acuity outcomes are similar across all procedures.

## Conclusion

TMH is the second most common cause of macular rupture, typically resulting from blunt ocular trauma. TMH cases are rarer than idiopathic macular holes, have distinct pathogenesis, and lack established treatment guidelines, particularly in pediatric patients. Some authors recommend 3 to 6 months of observation, especially in young patients with small ruptures and posterior vitreous adhesion at the TMH edges due to a higher chance of spontaneous closure. In cases with minimal likelihood of spontaneous closure, early pars plana vitrectomy with ILM peeling and gas tamponade appears to be a safe and effective treatment option for TMH in pediatric patients. Despite anatomical closure, functional outcomes may be limited by concurrent macular pathology induced by trauma. Compared to spontaneous closure rates reported in the literature, surgical closure has a higher success rate and yields better functional outcomes after a vitrectomy.
